# The Prevalence of Traditional Malpractice during Pregnancy, Child Birth, and Postnatal Period among Women of Childbearing Age in Meshenti Town, 2016

**DOI:** 10.1155/2018/5945060

**Published:** 2018-02-07

**Authors:** Haileyesus Gedamu, Adane Tsegaw, Etsubdink Debebe

**Affiliations:** School of Nursing, Bahir Dar University, Bahir Dar, Ethiopia

## Abstract

**Background:**

Cultural practices, beliefs, and taboos are often implicated in determining the care received by mothers during pregnancy and child birth which is an important determinant of maternal mortality.

**Objective:**

To assess prevalence of cultural malpractice during pregnancy, child birth, and postnatal period among women of childbearing age in Meshenti town, Amhara region, northwest Ethiopia, in 2016.

**Methods:**

Community based cross-sectional study was conducted among women of reproductive age group interviewed during the study period from May 10 to June 17, 2016. Total sample size was 318 women of reproductive age group. Systematic sampling technique was conducted.

**Result:**

Overall, 50.9% of the respondents had cultural malpractices during their pregnancy. Out of 318 women, 62 (19.5%) practiced nutrition taboo, 78 (24.5%) practiced abdominal massage, 87 (29.7%) delivered their babies at home, 96 (32.8%) avoided colostrums, 132 (45.2%) washed their baby before 24 hr after delivery, and 6 (6.9%) cut the cord by unclean blade.

**Conclusion and Recommendation:**

The findings of this study show that different traditional malpractice during perinatal period is still persisting in spite of modern developments in the world. Health education and promoting formal female education are important to decrease or avoid these cultural malpractices.

## 1. Introduction

Tradition represents the sum total of all behaviors that are learned, shared by a group of people, and transmitted from generation to generation. It includes language, religion, types of food eaten and methods of their preparation, childrearing practices, and all other values that hold people together and give them a sense of identity and distinguish them from other groups [1, 2].

Women all over the world are confronted with many difficult choices during pregnancy and child birth. Cultural practices, beliefs, and taboos are often implicated in determining the care received by mothers during pregnancy and child birth which is an important determinant of maternal mortality. Every day, at least 800 women die worldwide from the complications of pregnancy and child birth. These deaths are mainly due to obstetric problem which easily could be prevented if the mothers get the appropriate care they deserve during their pregnancy, delivery, and postnatal period [3, 4].

It has been recognized that violence and trauma, malpractice during pregnancy and childbirth, low social status, gender discrimination, and lack of awareness in ANC follow-up at nearby health facilities are direct and indirect medical causes of maternal mortality [5–7]. Harmful traditional practices that affect children and women are female genital mutilation, milk teeth extraction, food taboo, uvula cutting, forbidding food and fluids during diarrhea, keeping babies from exposure to sun, and feeding newborn babies with fresh butter [8, 9]. In Ethiopia, the great majorities of women deliver at home (90%) and follow the cultural birth customs. These create problems for several years associated with harmful cultural practices at each individual community and home during delivery [10, 11].

United Nations (UN) agencies and human right bodies started addressing harmful traditional practice in the early 1990s but there was little progress. There are now a number of important international instruments endorsed by most of the governments and could serve as a basis for a struggle against harmful traditional practice [12, 13]. In addition to deep-rooted beliefs, customs, and rational attitudes, lack of knowledge and being unaware of the effects of the practices help to maintain these problems. Sometimes a harmful practice is so deeply rooted that it seems impossible to change [14, 15].

In the world, there are many traditional malpractices during pregnancy, labor, and postnatal period. In Turkey and Iran, women use traditional practices to reduce engorgement of the breast and to increase the amount of breast milk [16–18].

In Guatemala, Guatemalan women believe speedy delivery can be induced by drinking a liquid created by boiling a purple onion in beer and in Niger, Muslim tradition allows only a woman's husband to touch her genitals, so midwives in this African nation facilitate labor by offering the mom-to-be herbal drinks and sprinkling herbs over her abdomen. Foods that are good sources of energy and protein are not allowed to be consumed by pregnant women for reasons such as difficult and prolonged labor due to fears of a large baby. Similarly, sources of vitamins and minerals are restricted during pregnancy mainly due to the fear of offensive discharges during delivery and skin diseases on the body. Studies conducted in central, eastern, and southern parts of Ethiopia have reported that food items that are white in color such as milk, fatty meat, porridge, potato, banana, clean vegetables, colostrums, and fruits are prohibited to be consumed by pregnant/lactating women and children [10, 19–21].

Another research done in Bangladesh on usage of colostrum and duration to initiate breastfeeding revealed that 90% of the mothers reported feeding their newborn colostrum. 59% of mothers initiated breastfeeding within 4 hours and 88% within 12 hours of parturition [22].

## 2. Methods and Materials

### 2.1. Study Area

The study was conducted in Meshenti town, west Gojjam zone, Amhara region, northwest Ethiopia. Meshenti was one of the towns found around Bahir Dar which has a total population of 6006, among those 2697 were males and 3309 were females. In addition, it has 1397 households and a total of reproductive age women (15–49 years) of 1418. The town has one health center and it has 1 Kebele; it is 555 km away from Addis Ababa and 12 km away from Bahir Dar.

Many harmful traditional practices have been performed in the study area. Therefore, this investigation revealed the evidence based occurrence of traditional malpractice during pregnancy, child birth, and postnatal period among women of child bearing age.

### 2.2. Study Design and Period

The community based cross-sectional study design was conducted from June 10 to June 17, 2016.

### 2.3. Source Population

The source population was all women of reproductive age group (15–49 years).

### 2.4. Study Population

The study population was all women in reproductive age (15–49 years) who experienced at least one pregnancy.

### 2.5. Inclusion and Exclusion Criteria

#### 2.5.1. Inclusion

All reproductive age women who experience at least one pregnancy were included.

#### 2.5.2. Exclusion

Women who had severe medical and mental illness and could not communicate verbally during data collection were excluded.

### 2.6. Sample Size Determination and Sampling Technique

#### 2.6.1. Sample Size Determination

The sample size was calculated by using the following single population proportion formula:

(1) $$\begin{array}{c} n={\left(\;z\;\right)}^{2}\times \frac{p\left(1-p\right)}{w^{2}}, \end{array}$$

where *z*^2^ is the confidence limit of the study (95%), *p* is the proportion of study population (38.3%), *w* is margin of error or desired precision (5% or 0.05), and *n* is total sample size.

Let us take *p* = 38.3% which is prevalence rate for home delivery which is most common traditional malpractice during delivery at Limmu Genet town, Oromia region, southwest Ethiopia [23].

The required sample size for this study “*n*” at confidence interval of 95% and marginal error of 5% will be determined by using population proportion formula: *n* = (1.96)^2^0.383(1 − 0.383)/(0.05)^2^ = 364. With correction formula *n*/1 + *n*/*N* (where *n* is the sample size we calculate as 364 and *N* is total population), our sample size becomes 289 and then, with 10% nonresponse rate, a total of 318 study participants were recruited.

#### 2.6.2. Sampling Technique and Procedure

The sampling technique was systematic random sampling which was conducted by considering list of households. The sampling interval was calculated using *k* = *N*/*n* (1397/318) = 4th, where *k* is sampling interval, *N* is number of households in Meshenti town, and *n* is sampling size.

The first sample was selected by using lottery method among the first four households and then assessed every 4th house. In households with no eligible person, we went to the next house. But for households with more than one eligible individual, one of the individuals was selected randomly.

### 2.7. Data Collection

Data was collected by using structured questionnaire by face-to-face interview. The questionnaire includes sociodemographic characteristics, reproductive health part, and traditional malpractice. Questionnaire that addressed the objective of the study was gathered and adapted. The questionnaire was prepared in English and translated to Amharic version to facilitate the understanding. Data collection was carried out by five data collectors from preparatory students trained on the study and data collection.

### 2.8. Data Quality Assurance

Pretest of questionnaires was done one week before the actual survey and it was carried out with similar study population in Merawi town for 1 day on 5% of sample size and the necessary modification was made before being applied on study subjects. The data was collected carefully by the trained data collector and principal investigators to get the reliable and necessary information according to the aim of the study. The principal investigator made an ongoing checkup each day during the data collection to ensure the quality of data by checking filled questionnaires for their completeness and internal consistency. The quality of data collection process was monitored by giving a clear attention to the data. All the collected data was checked and rechecked and we made the necessary correction each day; when there were difficulties occurring during the data collection, explanation and discussion with the respondents were done and unclear questions were explained briefly by using respondent native language.

### 2.9. Data Processing and Analysis

The collected data was entered and analyzed by using SPSS version 20. Calculation was calculated by using a calculator and Microsoft Excel and statistical analysis was done by principal investigator. Finally description, tables, graphs, and charts were used to present the finding.

## 3. Ethical Consideration

Ethical clearance was obtained from Bahir Dar University CMHS Ethical Review Board before data collection. Written and oral consent were obtained from the study population during data collection. The right was given for the study population to refuse or stop or withdraw at any time of data collection. The confidentiality of the respondents was ensured, where any person name did not appear on research documents and respondents were informed about the aim of the study.

## 4. Result

### 4.1. Sociodemographic Characteristics

A total of 318 participants were included in the study and response rate was 100%. Regarding the sociodemographic characteristics of the respondents, 100 (31.4%) were in the age group of 25–29. The majority of them were orthodox (252) (79.2%) and Amhara (314) (98.7%) by religion and ethnicity, respectively. Out of 318, 260 (81.8%) were married and 137 (41.1) were illiterate and 70 (22%) completed grades 1–8. Concerning occupation, 211 (66.4%) were housewives (Table 1).

### 4.2. Traditional Malpractices during Pregnancy

Generally in Meshenti town, 50.9% of pregnant women are involved in traditional malpractice during their pregnancy. Out of these, 62 (19.5%) had nutritional taboo, 139 (43.7%) used the mother's drink “telba” during their pregnancy, and 48 (15.1%) and 43 (13.5%) practiced abdominal massage and drank “kosso,” respectively (Table 2).

### 4.3. Traditional Malpractices during Child Birth

Generally in Meshenti town, 37.9% of mothers are involved in traditional malpractice during their child birth. From these, 6.3% of the mothers have traditional malpractice like abdominal massage and uterine massage and the other 29.7% have home delivery.

Out of 87 (29.7%) mothers who delivered their last child at home, most of them were assisted by their family (37) (42.5%), untrained traditional birth attendant (UTTBA) (29) (33.3%), neighbors (12) (13.8%), and trained traditional birth attendant (TTBA) (9) (10.3). Among 87 (29.7%) home deliveries, the cord was cut by using unboiled new blade (66) (75.9%), boiled new blade (14) (16.1%), unboiled blade used before (6) (6.9%), and boiled blade used before (1) (1.1%). From the 87 home deliveries, 84 of them had their umbilical cord tied using boiled thread (71) (84.5%), unboiled thread (10) (11.9%), and other material (3) (3.6%) (Table 3).

**Figure 1:**
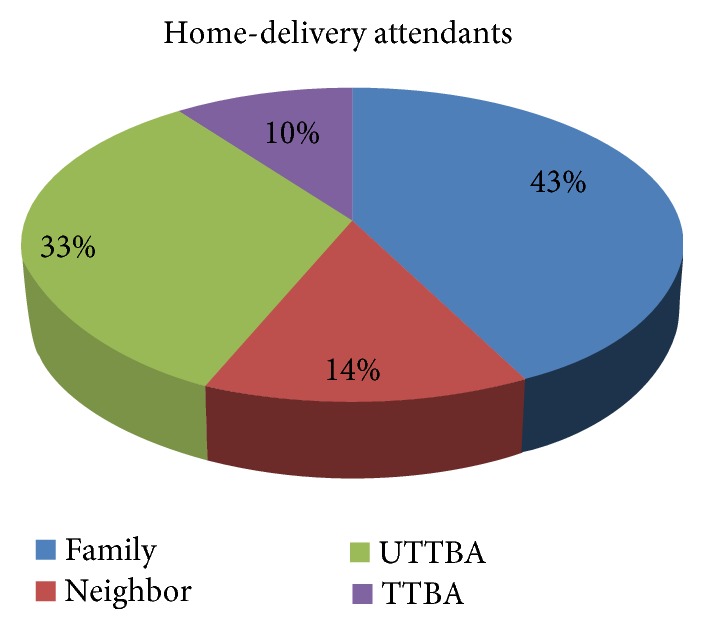
Distribution of birth attendants for women who deliver at home in Meshenti town, northwest Ethiopia, Amhara region, June 2016.

For home-delivery mothers, the majority (43%) of them were assisted by their family whereas 10% of them were assisted by traditional trained birth attendants (Figure 1).

### 4.4. Traditional Malpractices during Postnatal Period

Generally in Meshenti town, 76.1% of the mothers are involved in traditional malpractice during their postnatal period. From these 293 mothers, 96 (32.8%) mothers avoided colostrum and 98 (30.4%) of the mothers applied butter on their child umbilicus after delivery. some of the respondents started breastfeeding within 24 hr and 23 (7.8%) after 24 hr. On the other hand, 81 (27.6%) mothers washed their babies immediately after delivery (Table 4).

**Figure 2:**
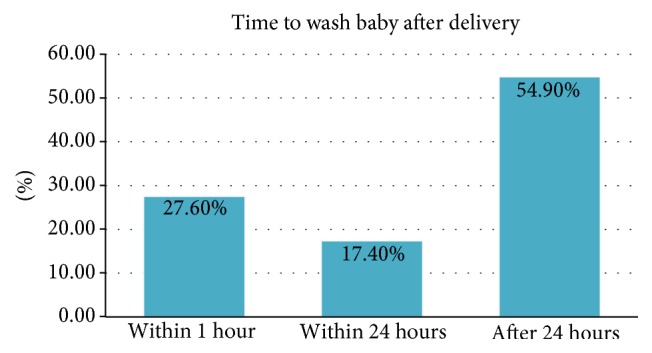
Time period of washing babies after delivery among reproductive aged women in Meshenti town, west Gojjam, northwest Ethiopia, June 2016.

The majority of babies delivered at Meshenti town had their bodies washed after 24 hrs (Figure 2).

## 5. Discussion

This community based study has attempted to assess the prevalence of traditional malpractices during pregnancy, delivery, and postnatal period in Meshenti town, west Gojjam zone, Amhara region, northwest Ethiopia. The result of the study revealed that, from the total of 318 respondents, 100 (31.4%) of the women were of the age range of 25–29 years and majority were married and illiterate: 260 (81.8%) and 137 (43.1%), respectively. This result was in line with a research done at north Arbaminch, south Ethiopia, where 36% of the women were aged 25–29 and 90.8% and 53.6% were married and illiterate, respectively [23].

Our study reveals that the prevalence of nutritional taboo was 19.5%. This result was slightly similar to a study conducted in Lemmu genet town with prevalence of 19.1% [24]. The result was also lower than that of a study conducted in Shashemene with the prevalence of 49.8% [21]. The possible reason for the variation of the results was different characteristics in study population. The food items that are avoided during pregnancy, based on our research, are vegetables (6 times), meat (2 times), paper (34 times), “genfo” (9 times), sugar cane (16 times), and “ergo” (7 times); a research done in Shashemene shows vegetables (17 times) and meat (63 times) which is very high [21]. The major reasons for avoiding these food items during pregnancy were that the food may plaster the fetal head or make the baby fat and difficult to deliver.

As Guatemala women believe that drinking liquid (which is prepared by boiling purple onion in beer) is very helpful to induce labor, our research also shows that Meshenti women drink telba to facilitate their labor [19].

Our study revealed that the prevalence of home delivery was 29.7% which was lower than that of the study conducted in Lemmu genet town, Oromia region, Jimma zone, 38.3% [24]; however, our study was comparable with a study conducted in south Gondar zone, Debretabor town, where the prevalence was 29.6% [25]. Out of 29.7% of the home delivered babies, most of them were attended by family (42.5%) and untrained traditional birth attendants (33.3%) which is higher and lower than the study conducted in Debretabor town, south Gondar zone, 13%, and south Arbaminch, north Ethiopia, 79.4%, respectively [23, 25].

This investigation also revealed that prevalence of avoiding colostrum was 32.8% which was lower than study conducted in Bangladesh with the prevalence of 41% [22]; and the result was lower than the study conducted in Raya Kobo district, northeastern Ethiopia, with the prevalence of 13.5% [26]. In this study, 132 (45%) of the mothers washed their babies before 24 hours after delivery that is higher than the research done at Lemmu genet town, Oromia region, Jimma zone, which was 28.4% [24]. This may cause negative impact on the newborn baby like hypothermia.

About 6.9% of respondents used unsterile material to cut the umbilical cord during home delivery which was higher than the study conducted in South Gondar zone, Debretabor town, which was 0.6% [25].

A study conducted at Debretabor governmental health institution shows that 213 (60%) have started breastfeeding within one hour, 109 (30.7%) within 24 hr, and 33 (9.3%) after 24 hours (d). But our study revealed that 219 (74.7%) have started breastfeeding within one hour, 51 (17.4%) within 24 hr, and 23 (7.8%) after 24 hours [25].

Based on our study, 24.5% (78) of the mothers had abdominal massage during pregnancy which was relatively similar to a research done at Lemmu genet town, Oromia region, Jimma zone, with prevalence of 22% [24] and higher when compared to a research done at Debretabor governmental health institution, south Gondar zone, Debretabor town, 12.1% [25]. The possible reason to do such a practice is to avoid striae and to relieve back pain that occurred during pregnancy.

## 6. Conclusion

The prevalence of cultural malpractice in the study area was found to be high. Therefore, health education focusing on problems of cultural malpractice is recommended to reduce or avoid the consequences of the malpractice.

## Figures and Tables

**Table 1:** Sociodemographic characteristics of respondents among women of childbearing age in Meshenti town, Bahir Dar Zuria woreda, Amhara region, June 2016.

Characteristics	Category	Frequency	Percent (%)
Age	15–19	15	4.7
	20–24	89	28.0
	25–29	100	31.4
	30–34	50	15.7
	35–39	34	10.7
	40–44	14	4.4
	45–49	16	5.0
	*Total*	*318*	*100.0*

Religion	Orthodox	252	79.2
	Muslim	64	20.1
	Protestant	2	.6
	*Total*	*318*	*100.0*

Ethnicity	Amhara	314	98.7
	Oromo	2	.6
	Tigre	2	.6
	*Total*	*318*	*100.0*

Educational status	Have no formal education	172	44.1
	G 1–8	70	22.0
	G 9–12	53	16.7
	12+	23	7.2
	*Total*	*318*	*100.0*

Marital status	Married	260	81.8
	Single	18	5.7
	Divorced	24	7.5
	Widowed	16	5.0
	*Total*	*318*	*100.0*

Occupational status	Housewife	211	66.4
	Employee	25	7.9
	Merchant	45	14.2
	Student	6	1.9
	Other	31	9.7
	*Total*	*318*	*100.0*

**Table 2:** Traditional malpractice during pregnancy in Meshenti town, 2016.

Variables	Frequency	Percent (%)
Nutritional taboos	62	19.5
Abdominal massage	48	15.1
Drinking telba	139	43.7
Drinking kosso	43	13.5
Abdominal massage and drinking telba	20	6.3
Abdominal massage and drinking kosso	10	3.1
Drinking telba and drinking kosso	1	.3
Other	6	1.9
I don't know	51	16

**Table 3:** Traditional malpractice during delivery in Meshenti town, 2016.

Variables	Frequency	Percent (%)
Traditional malpractice		
Abdominal massage		
Yes	8	2.73
No	0	0
Uterine massage		
Yes	7	2.39
No	0	0
Abdominal and uterine massage		
Yes	5	1.7
No	0	0
*Total*	*20*	*6.82*
Place of delivery		
Home	87	29.7
Health facility	206	70.3
*Total *	*293*	*100*
Method used to expel placenta		
Spontaneously	76	87.4
Uterine massage	11	12.6
*Total *	*87*	*100*
Instrument used to cut cord		
Boiled new blade	14	16.1
Unboiled new blade	66	75.9
Unclean blade used before	6	6.9
Boiled blade used before	1	1.1
*Total*	*87*	*100*
Umbilical cord tied		
Yes	84	96.6
No	3	3.4
*Total *	*87*	*100*
Material used to tie umbilical cord		
Boiled thread	71	84.5
Unboiled thread	10	11.9
Other	3	3.6
*Total *	*84*	*100*

**Table 4:** Traditional malpractice during postnatal period in Meshenti town, 2016.

Variables	Frequency	Percent (%)
What was applied on the stump of umbilicus		
Nothing	174	54.4
Cow dung	4	1.4
Butter	98	30.4
Other	17	5.8
*Total *	*293*	*100*
What was given to the child immediately after delivery		
Butter	60	20.5
Cow milk	5	1.7
Water	7	2.4
Nothing	210	71.7
Other	11	3.8
*Total *	*293*	*100*
Time to start breastfeeding		
Within 1 hour	219	74.7
Within 24 hours	51	17.4
After 24 hours	23	7.8
*Total *	*293*	*100*
Avoiding colostrum		
Yes	197	67.2
No	96	32.8
*Total *	*293*	*100*

## References

[1] Asefa D., Wassie E., Getahun M., Berehaneselassie M., Melaku A. (2005). *Harm Full Traditional Practices for the Ethiopian Health Center Team*.

[2] CSA (2005). *Ethiopia Demographic and Health Survey*.

[3] UNCHR (2006). *Harmful Traditional Practices Affecting the Health of Women and Children*.

[4] WHO (2014). *Maternal Mortality*.

[5] Raelori J. Cultural child birth practice’s, believes & tradition in Libya, 2009.

[6] Adolescent pregnancy cultural complex issue

[7] Otoo P., Habib H., Ankomah A. (2015). Food prohibitions and other traditional practices in pregnancy: a qualitative study in western region of ghana. *Advances in Reproductive Sciences*.

[8] MHRC (2005). *Cultural Practices and their impact on the enjoyment of human rights, Particularly the Rights of Women and Children in Malawi*.

[9] Dawit A., Eshetu W., Masresha G., Misganaw B., Atsinaf M. (2004). *Harmful Traditional Practices: Module for Ethiopian Health Center Team*.

[10] Neguse M., Hailemariam D., Metike G. (2004). Assessment of safe delivery services utilization among WCBA North Gondar zone, North West Ethiopia. *Ethio journal health*.

[11] EGLDAM (2008). *Follow up National Survey on Harmful Traditional Practices in Ethiopia*.

[12] Zuberi M.

[13] Alene G. D., Edris M. (2002). Knowledge, attitudes and practices involved in harmful health behavior in Dembia district, Northwest Ethiopia. *Ethiopian Journal of Health Development*.

[14] Worku F., Gebresilassie S. (2008). *Reproductive Health for Health Science Students*.

[15] WHO (2008). *WHO Regional Health Forum South-East Asia Region*.

[16] https://www.ncbi.nlm.nih.gov/pubmed/8693724

[17] Suheyla A., Katabi V. Comparison of Cultural Practices Used in Pregnancy Postpartum Periods among Women in Turkey & Iran.

[18] McKinley A. Traditions from around the world; from conception to birth and beyond, ancient traditions and cultural beliefs have always shaped the art of motherhood. https://www.pnmag.com.

[19] Ethiopian public health training initiative (EPHTI) (2003). *Pediatrics & Child Health Lecture Note for Health Science Students*.

[20] Zepro N. (2015). Food taboos and misconceptions among pregnant women of shashemene district, ethiopia. *Science Journal of Public Health*.

[21] Holman D. J., Grimes M. A. (2001). Colostrum feeding behaviour and initiation of breast-feeding in rural Bangladesh. *Journal of Biosocial Science*.

[22] Ayele G. (2015). Prevalence and associated factors of home delivery in arbaminch zuria district, southern ethiopia: community based cross sectional study. *Science Journal of Public Health*.

[23] Tola T., Tadesse A. (2015). Cultural malpractices during pregnancy, child birth and postnatal period among women of child bearing age in limmu genet town, southwest Ethiopia. *Science Journal of Public Health*.

[24] Zenebe K., Alem H., Merga A., Abate G., Taha H. (2015). prevalence of cultural malpractice and associated factors among women attending mch clinic at debretabor governmental health institution south gondar, amhara region, north west Ethiopia. *Gyneco and Obstet*.

[25] Legesse M., Demena M., Mesfin F., Haile D. (2015). Factors associated with colostrum avoidance among mothers of children aged less than 24 Months in Raya Kobo district, North-eastern Ethiopia: Community-based cross-sectional study. *Journal of Tropical Pediatrics*.

[26] Rowen T., Prata N., Passano P. (2011). Evaluation of a traditional birth attendant training programme in Bangladesh. *Midwifery*.

